# Structural insight into ASH1L PHD finger recognizing methylated histone H3K4 and promoting cell growth in prostate cancer

**DOI:** 10.3389/fonc.2022.906807

**Published:** 2022-08-10

**Authors:** Miaomiao Yu, Yanjie Jia, Zhanchuan Ma, Donglei Ji, Chunyu Wang, Yingying Liang, Qiang Zhang, Huanfa Yi, Lei Zeng

**Affiliations:** ^1^ Bethune Institute of Epigenetic Medicine, The First Hospital, Jilin University, Changchun, China; ^2^ International Center of Future Science, Jilin University, Changchun, China; ^3^ Central Laboratory, The First Hospital, Jilin University, Changchun, China; ^4^ State Key Laboratory of Supramolecular Structure and Materials, College of Chemistry, Jilin University, Changchun, China

**Keywords:** ASH1L, PHD finger, histone methylation, H3K4me2, prostate cancer, cell cycle and apoptosis

## Abstract

ASH1L is a member of the Trithorax-group protein and acts as a histone methyltransferase for gene transcription activation. It is known that ASH1L modulates H3K4me3 and H3K36me2/3 at its gene targets, but its specific mechanism of histone recognition is insufficiently understood. In this study, we found that the ASH1L plant homeodomain (PHD) finger interacts with mono-, di-, and trimethylated states of H3K4 peptides with comparable affinities, indicating that ASH1L PHD non-selectively binds to all three methylation states of H3K4. We solved nuclear magnetic resonance structures picturing the ASH1L PHD finger binding to the dimethylated H3K4 peptide and found that a narrow binding groove and residue composition in the methylated-lysine binding pocket restricts the necessary interaction with the dimethyl-ammonium moiety of K4. In addition, we found that the ASH1L protein is overexpressed in castrate-resistant prostate cancer (PCa) PC3 and DU145 cells in comparison to PCa LNCaP cells. The knockdown of ASH1L modulated gene expression and cellular pathways involved in apoptosis and cell cycle regulation and consequently induced cell cycle arrest, cell apoptosis, and reduced colony-forming abilities in PC3 and DU145 cells. The overexpression of the C-terminal core of ASH1L but not the PHD deletion mutant increased the overall H3K36me2 level but had no effect on the H3K4me2/3 level. Overall, our study identifies the ASH1L PHD finger as the first native reader that non-selectively recognizes the three methylation states of H3K4. Additionally, ASH1L is required for the deregulation of cell cycle and survival in PCas.

## Introduction

Histone methylations are critical for epigenetic regulation and gene expression (1, 2). They play an important role in virtually all biological processes, from DNA repair, cell cycle, stress response, and transcription to development, differentiation, and aging (3, 4). In general, the methylation of histone H3 lysine 4 (H3K4) and lysine 36 (H3K36) is used for gene transcription activation (1, 5, 6), whereas the methylation of H3K9 (7) and H3K27 (8) is associated with gene repression (1). H3K4 methylation is an evolutionarily conserved histone modification. Dimethylated histone H3 lysine 4 (H3K4me2) and H3K4me3 were shown to facilitate epigenetic reader protein recruitment to chromatin (9, 10). High levels of H3K4me1, H3K4me2, and H3K4me3 are detected in the promoters and on both sides of transcription start sites (1, 11–13); H3K4me2, H3K4me3, and H3K27ac are enriched at active enhancers (14–17), whereas H3K4me1 can be associated with both active and inactive enhancers (16–18). Therefore, the identification of the recognition profiles of these H3K4 methylation marks may help to understand the regulatory elements of the underlying genes and their epigenetic regulation.

**Figure 1:**
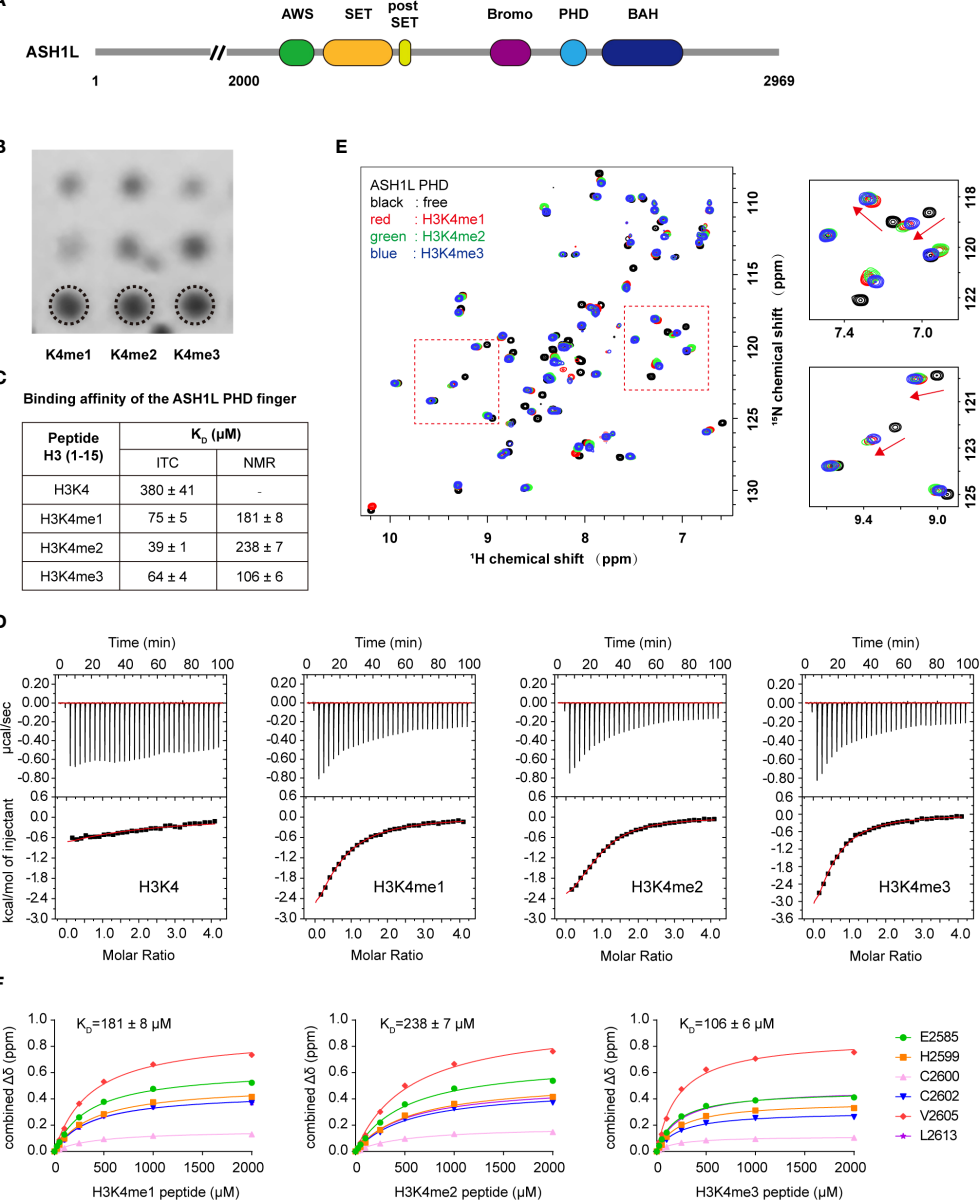
ASH1L PHD finger shows comparable binding affinity to H3K4me1/me2/me3. **(A)** Schematic representation of ASH1L protein. **(B)** Histone peptide array probed with the His-tagged ASH1L PHD finger. **(C)** Binding affinity of ASH1L PHD for the indicated H3 (1–15) peptides measured by isothermal titration calorimetry (ITC) and NMR titration. **(D)** Representative binding carves used to determine the K_D_ values by ITC. **(E)** Superimposed ¹H,^15^N HSQC spectra of the ASH1L PHD finger collected upon titration with the indicated peptides in a protein–peptide molar ratio of 1: 5. Spectra are color-coded according to different peptides. Arrows indicate chemical shift changes. **(F)** Representative saturation curves used to determine the K_D_ values by NMR titration. The saturation curves were calculated using a single-site binding equilibrium model.

ASH1L (absent, small, or homeotic disc1-like) is a member of the Trithorax-group (TrxG) protein (19, 20). ASH1L consists of an AWS (associated with SET) domain, an SET domain, a cysteine-rich post-SET domain, a bromodomain (BrD), a plant homeodomain (PHD) zinc finger, and a bromo-adjacent homology (BAH) (21) (**Figure 1A**). The conserved SET domain harbors lysine methyltransferase activity (22). ASH1L usually localizes in different cellular compartments, such as small speckles in the cell nucleus and cell–cell tight junctions (23). It promotes gene expression by methylating histone H3K4 and H3K36 and counteracts gene silencing mediated by the Polycomb group protein (24–26). In mammals, ASH1L plays important roles in the normal development and function of the nervous system (27–29), muscular dystrophy pathogenesis (30, 31), and immune cell development (23, 32, 33). *ASH1L* is also an emerging oncogene in acute leukemia (34, 35), thyroid carcinoma (36, 37), prostate cancer (PCa) (38), renal cell carcinoma (39), and hepatoma (40). However, the first-in-class inhibitor of the ASH1L SET domain, AS-99, has shown remarkable anti-leukemic activity (34) against MLL1 fusion leukemia, suggesting that ASH1L can be potentially targeted for the therapeutic cancer treatment.

PHD zinc fingers are structurally conserved modules found in many nuclear proteins including transcription factors, histone-modifying enzymes, and ATP-dependent chromatin remodeling complexes and plays an important role in recognizing chromatin modifications and recruiting regulatory proteins to specific genes (41–43). A subset of PHD fingers has been shown to bind the histone lysine residues of different modification states, such as H3K4me0, H3K4me3, H3K9me3, and H3K14ac (44). For instance, the PHD fingers of MLL5 (mixed-lineage leukemia 5) (45), ING2 (inhibitor of growth 2) (46), and ING3 (47) have a binding preference to H3K4me3 rather than H3K4me2; the Set3 PHD finger shows similar binding affinities to H3K4me2 and H3K4me3 (48); and the PHF20 PHD selectively recognizes H3K4me2 (49). Notably, the PHD fingers of BHC80 (50) and AIRE (51) prefer to bind unmethylated H3K4 (H3K4me0), and this interaction is completely abolished, following the methylation of H3K4 (50, 51). Histone H3K4 methylation may also recruit TrxG proteins by providing docking sites for specific ‘reader’ modules. For example, the third PHD finger of MLL1 directly recognizes H3K4me2 and H3K4me3 and is necessary for MLL1 recruitment to the *HOX9A* gene locus and activation of the gene transcription in leukemogenesis (52). The NURF301-BPTF PHD finger can selectively read H3K4me3, thereby linking H3K4me3­mediated gene activation with NURF-mediated ATP-dependent chromatin remodeling (43). However, it is still not completely understood how the ASH1L PHD finger binds methylated H3K4 and functions as a platform for ASH1L in biological processes.

In this study, we report that the ASH1L PHD finger acts as a non-selective histone H3K4 methylation reader. The solution nuclear magnetic resonance (NMR) structure of the PHD domain of ASH1L in complex with the H3K4me2 peptide reveals a narrow binding cage and unique residue composition, which enable the ASH1L-PHD to bind all three methylation states of H3K4 peptides with comparable affinities. In addition, ASH1L is more highly expressed in castrate-resistant PCa (CRPC) PC3 and DU145 cells than in PCa LNCaP cells. The knockdown of ASH1L modulates gene expression and cellular pathways involved in apoptosis and cell cycle regulation and consequently induces cell cycle arrest, cell apoptosis, and reduced colony-forming abilities in PC3 and DU145 cells. The overexpression of the C-terminal core of ASH1L but not the PHD deletion mutant increased the overall H3K36me2 level but had no effect on the H3K4me2/3 level, suggesting that the deletion of the PHD domain likely abolished the recruitment of ASH1L to chromatin and consequently suppressed ASH1L-mediated H3K36 methylation.

## Materials and methods

### Plasmid construction

The mASH1L PHD domain (residue 2573–2624) was subcloned into a modified pMAL-c5X expression vector (New England Biolabs, Ipswich, MA, US), which contains an MBP tag and a TEV cleavage site. The site-directed mutagenesis of D2573A, I2575A, D2584E, E2585A, M2588A, Q2590E, and W2597A in the PHD domain was generated using the *PfuTurbo* DNA polymerase (Agilent Technologies) and Dpn I restriction enzyme (New England Biolabs). After the MBP tag was cut by TEV, these proteins were used in ITC and NMR experiments. The His-tagged PHD domain was subcloned into a pGEX-4T1 expression vector. The base vector has a GST tag and was engineered to include a TEV cleavage site before a 5′ 6×His sequence. After the GST tag was cut, this protein was used for the peptide array assay. The His-tagged Bromo-PHD tandem domain (residue 2425–2627) of mASH1L was subcloned into a pNIC-Bsa4 expression vector, which contains a His tag. For the ASH1L overexpression plasmids, the C-terminal construct of hASH1L (Flag-ASH1L-C, residue 1892–2969) was amplified by PCR, and the C-terminal construct with PHD domain deletion (Flag-ASH1L-C-ΔPHD) (residue 1892–2969 with 2585–2636 deletion) was generated by overlapping PCR. The two constructs were cloned into a pCMV-Flag-7.1 eukaryotic expression vector. All plasmids were confirmed by DNA sequencing.

### Protein expression and purification

The bacteria were grown in Luria Broth (LB) media, supplemented with 100-μM ZnCl_2_ (all PHD constructs). All PHD domains were expressed in *Escherichia coli* BL21 (DE3) cells and induced overnight with 0.5-mM isopropyl-β-D-1-thiogalactopyranoside (IPTG) at 22°C. In addition, the cells were harvested by centrifugation at 4,000 g. The harvested cells with MBP-tagged PHD were resuspended in buffer containing 25-mM Tris, pH 7.2, 1-M NaCl, 20-mM β-mercaptoethanol, 100-μM ZnCl_2_, and the Complete EDTA-free Protease Inhibitor (Roche, Mannheim, Germany). Following lysis in a microfluidizer (MFIC), cell debris was removed by centrifugation at 12,000 g for 1 h and the supernatant was loaded on a 5-ml MBPTrap HP column (GE Healthcare). The binding protein was eluted with buffer containing 25-mM Tris, pH 7.2, 500-mM NaCl, 20-mM β-mercaptoethanol, 20-mM maltose, and 100-μM ZnCl_2_. After TEV cleavage, the PHD domain was supplemented with 20-mM DTT and further purified using a Superdex 75 column (GE Healthcare, Uppsala, Sweden) in a final buffer containing 1×PBS, pH 7.2, 100- μM ZnCl_2_. The ASH1L-PHD mutant proteins were prepared as described above. For the NMR study, the ^15^N or ^13^C-labeled ASH1L-PHD proteins were expressed by growing the bacteria in a ^15^NH_4_Cl- or ^13^C-glucose-enriched M9 minimal medium and then purified as unlabeled proteins. The His-tagged PHD protein was expressed in a GST tag containing a vector. The lysis buffer was 1×PBS, pH 7.2, 500-mM NaCl (total), 5% glycerol, 100-μM ZnCl_2_, 0.2% NP-40, and Complete EDTA-free Protease Inhibitor. The protein was purified with a 5-ml GSTrap HP column (GE Healthcare, Uppsala, Sweden) and eluted with buffer containing 50-mM Tris, pH 7.2, and 20-mM glutathione. After the GST tag was cleaved by TEV protease, the His-tagged PHD domain was further purified with the Superdex 75 column in the same final buffer containing 1×PBS, pH 7.2, 100-μM ZnCl_2_. The size and purity of the purified protein were monitored by SDS-PAGE. The PHD protein was concentrated with a filter tube (Millipore, Co. Cork, Ireland) and measured by UV absorbance at 280 nm.

The His-tagged Bromo-PHD tandem protein was expressed in a His tag-containing vector. The lysis buffer was 30-mM HEPES, pH 7.2, 500-mM NaCl, 10-mM imidazole, 10% IGEPAL, and the Complete EDTA-free Protease Inhibitor (Roche, Mannheim, Germany). The protein was purified with a 5-ml HiTrap IMAC FF column (GE Healthcare, Uppsala, Sweden) and eluted with buffer containing 30-mM HEPES, pH 7.2, 500 mM NaCl, 250-mM imidazole, 5% glycerol, and 0.1% CHAPS (3-((3-cholamidopropyl) dimethylammonio)-1-propanesulfonate). The His-tagged Bromo-PHD tandem domain was further purified with the Superdex 75 column in the same final buffer containing 1×PBS, pH 7.2, 100-μM ZnCl_2_.

### Isothermal titration calorimetry

ITC assays were carried out on the MicroCal iTC200 System (Malvern) at 25°C while stirring at 750 rpm in the ITC buffer of pH 7.2, consisting of 1×PBS, 100-μM ZnCl_2_. The histone H3 (1–15) peptides with un-, mono-, di-, and trimethylation modification on lysine 4 (H3K4, H3K4me, H3K4me2, H3K4me3), H3 (2–16) peptide with dimethylation on lysine 9 (H3K9me2), and H3 (29–43) peptide with dimethylation on lysine 36 (H3K36me2) were synthesized by MIMOYOPES (Wuxi, China). The PHD or tandem Bromo-PHD protein sample of 100 µM was placed in the cell and titrated with 3 mM of a different peptide solution in the ITC buffer using a set of 31 injections of 1.2 μl, following a single initial injection of 0.2 μl. In the ITC measurements of PHD mutant samples, the H3 (1–15) peptides were diluted to the final concentrations of 1.5 mM, titrated with a single initial injection of 0.4 μl and followed with a set of 19 injections of 2 μl. Cumulative enthalpy exchange plots were derived from the raw thermal changes, using one set of a site-fitting model. The thermodynamic parameters and binding constants were taken from the analysis done by Origin 7 SR4 (v7.0552) (OriginLab, Northampton, MA, US) provided with MicroCal iTC200.

### Peptide array assay

The peptide array assay was performed using the MODfied Histone Peptide Array Kit (Active Motif, Carlsbad, CA, US, catalog no. 13005), which contains an array of histone-modified polypeptides with different modifications and permutations in 384 dot matrixes. Each kit is divided into left and right wings and a repeating lattice arrangement. The array was first immersed in a 5% non-fat powdered milk (Sangon Biotech, Shanghai, China) blocking solution and incubated on an orbital shaker for overnight at 4°C. The next day, the array was quickly rinsed with TTBS buffer (10-mM Tris-HCl, pH 7.4, 150-mM NaCl, and 0.05% Tween20) and washed three times for 5 min each. Then, 3 ml of His-tagged PHD (30 μM) in protein buffer (1×PBS, pH 7.2, 100-μM ZnCl_2_) was incubated with the blocked array overnight at 4°C. Next, the array was washed again with TTBS buffer three times and then incubated with the anti-His tag primary Mouse monoclonal antibody (Sangon Biotech, Shanghai, China, catalog no. D191001-0200) overnight at 4°C. The same washing procedure was repeated, and the array was incubated with the secondary antibody HRP-conjugated goat anti-Mouse IgG (Sangon Biotech, Shanghai, China, catalog no. D110087-0025) for 2 h at room temperature. After the same washing procedure, the signal was detected on the Azure c500 Gel Imaging System (Azure Biosystems) using a PiercecECL Western Blotting Substrate (Thermo Fisher Scientific, Rockford, IL, US, catalog no. 34095). The results were analyzed by the corresponding array analysis software.

### Plant homeodomain finger-binding study by nuclear magnetic resonance

The ^1^H,^15^N HSQC spectra of the ^15^N-labeled PHD finger were collected at 298 K on a Bruker 800-MHz NMR spectrometer equipped with the z-gradient triple-resonance cryoprobe. The binding was analyzed through monitoring chemical shift perturbations observed in the two-dimensional ^1^H,^15^N HSQC spectra of PHD finger upon the addition of H3 (aa 1-15) peptides (H3K4me, H3K4me2, H3K4me3). The ^15^N-labeled PHD finger (0.05 mM) in complex with peptides (0.25 mM) were prepared in 1×PBS, pH 7.2, 100-μM ZnCl_2_ buffer in H_2_O/^2^H_2_O (9:1). The molar ratio of protein to peptides was 1:5. The bindings of the H3K9me2 (aa 2-16, 0.5 mM) or H3K36me2 (aa 29-43, 0.5 mM) peptide with the ^15^N-labeled PHD finger (0.1 mM) were analyzed with the same buffer condition.

### K_D_ estimation by nuclear magnetic resonance

The PHD domain at a concentration of 100 μM was titrated using different methylated H3K4 peptides at an increasing concentration ranging between 25 and 2,000 μM. The free form was used as the reference. The buffer and pH conditions are the same as above. The weighted chemical shift difference (Δ*δ*_weighted_) was calculated using the equation: $\Delta \delta_{\text{weighted}}=\sqrt{{\left|\Delta \delta \text{H}\right|}^{2}+{\left|\Delta \delta \text{N}\right|}^{2}*0.15}$ , where ΔδH is the chemical shift on the proton and ΔδN is the chemical shift on the nitrogen that is scaled with a factor of 0.15 to account for the different range of the amide proton and amide nitrogen. Chemical shifts for each backbone amide group were measured from the peak detected in the free-form spectrum to the peak at the end of the titration. K_D_ for each peptide was estimated using the following equation: $\Delta \delta_{\text{obs}}=\Delta \delta_{\text{max}}\frac{\left\{{\text{K}}_{\text{d}}+\text{Pt}+\text{Lt}-\sqrt{{\left({\text{K}}_{\text{d}}+\text{Pt}+\text{Lt}\right)}^{2}-4\text{Pt}*\text{Lt}}\right\}}{2\text{Pt}}$ , where [P]t and [L]t are the total concentration of protein and ligand, Δδ_obs_ is the observed chemical shift change regarding the reference, and Δδ_max_ is the maximum chemical shift change at saturation (53). K_D_ for each peptide was extrapolated as an average value of mean ± SEM.

### Nuclear magnetic resonance spectroscopy

The ASH1L PHD finger/H3K4me2 peptide (1–15) complex was used for structure determination. The NMR samples of the PHD finger (0.5 mM) in complex with an H3K4me2 peptide (ARTKme2QTARKSTGGKA) of 2.5 mM were prepared in buffer containing 1×PBS, pH 7.2, and 100-μM ZnCl_2_ in H_2_O/^2^H_2_O (9/1) or ^2^H_2_O. All NMR spectra were acquired at 298 K on a Bruker 800-MHz NMR spectrometer equipped with the z-gradient triple-resonance cryoprobe. The backbone ^1^H, ^13^C, and ^15^N resonances were assigned using standard three-dimensional (3D) triple-resonance HNCA, HN(CO)CA, HN(CA)CB, and HN(COCA)CB experiments (54). The side-chain atoms were assigned from 3D HCCH-TOCSY, HCCH-COSY, and (H)C(CO)NH-TOCSY data (55). The NOE-derived distance restraints were obtained from ^15^N- or ^13^C-edited 3D NOESY spectra. The H3K4me2 peptide was assigned from two-dimensional TOCSY, NOESY, ROESY, and ^13^C/^15^N-filtered TOCSY and NOESY. The intermolecular NOEs used in defining the structure of the complex were detected in ^13^C-edited (F1), ^13^C/^15^N-filtered (F3) 3D NOESY spectra (56). Spectra were processed with NMRPipe and analyzed using NMRview (57, 58).

### Structure calculations

The structures of the ASH1L PHD finger/H3K4me2 peptide were calculated with a distance-geometry simulated annealing protocol with CNS (59). Initial protein structure calculations were performed with manually assigned NOE-derived distance constraints. The hydrogen bond distance and Φ and ψ dihedral-angle restraints from the TALOS-N prediction were added at the later stage of structure calculations for residues with characteristic NOE patterns (60, 61). The converged structures were used for the iterative automatic NOE assignment by ARIA refinement. Structure quality was assessed with CNS, ARIA, and PROCHECK analysis (62, 63).

### Cell culture and small interfering RNA or overexpression plasmid transfection

PC3 and DU145 cell lines were cultured in RPMI 1640 (Sigma-Aldrich, St. Louis, MO, US) supplemented with 10% fetal bovine serum (Biological Industries, Kibbutz Beit Haemek, Israel) and 1% Pen Strep (Gibco, Grand Island, NY, US). All cell lines were cultured in an atmosphere of 5% carbon dioxide at 37°C. The human-specific siRNAs targeting ASH1L were designed and synthesized by GenePharma (Shanghai, China). The sequences of siRNA were as follows: negative control siNC 5’-UUCUCCGAACGUGUCACGUTT-3’; ASH1L siRNA#1 5’-GCUGAAAGCCUAAGUACUATT-3’; ASH1L siRNA#3 5’-CCAGCCAACCUCUGAUAAATT-3’. PC3 and DU145 cells were seeded in 12- or 6-well plates. After 24 h, siRNA was transfected with a TransIntro™ EL Transfection Reagent (TransGen Biotech, Beijing, China, catalog no. FT201) according to the manufacturer’s instruction. The siNC was used as negative-control siRNA. For the overexpression assay, the overexpression plasmids were transfected into 293T cells by polyethyleneimine (PEI).

### Cell lysis and immunoblotting

Cells were lysed with RIPA buffer (Solarbio, Beijing, China, catalog no. R0020) containing PMSF (Solarbio, Beijing, China, catalog no. P0100), Proteinase Inhibitor Cocktail (TransGen Biotech, catalog no. DI101-01), and PhosSTOP (Roche, Mannheim, Germany, catalog no. 4906837001). The total protein concentration was determined by the Enhanced BCA Protein Assay Kit (Beyotime Biotechnology, Shanghai, China, catalog no. P0010). These cell extracts were mixed with 4×Protein SDS-PAGE loading buffer (Takara Bio, catalog no. 9173), and heated at 104°C for 10 min. The same amount of cell extract was loaded and analyzed *via* immunoblotting using the standard protocol. The following antibodies were used: anti-GAPDH (BBI, Shanghai, China, catalog no. D110016-0200), anti-ASH1L (Santa Cruz Biotechnology, Dallas, TX, US, catalog no. sc-81052), anti-ASH1L (Abcam, Cambridge, UK, catalog no. ab5098), anti-H3K4me2/3 (Abcam, catalog no. ab6000), anti-H3K36me2 (Abcam, Cambridge, UK, catalog no. 176921), anti-Flag (Sigma, St. Louis, MO, US, catalog no. F1804), anti-caspase3 (Zen Bioscience, Chengdu, China, catalog no. 341034), anti-cleaved PARP (Cell Signaling Technology, Danvers, MA, US, catalog no. 5625), anti-Cyclin A1 (Abcam, Cambridge, UK, catalog no. 270940), anti-cyclin D1 (Proteintech Group, catalog no. 60186-1-Ig), HRP-conjugated anti-mouse IgG (BBI, catalog no. D110087-0025), HRP-conjugated anti-rabbit IgG (Jackson ImmunoResearch, West Grove, PA, US, catalog no. 111-035-003). Proteins were detected using the SuperSignal West Femto Maximum Sensitivity Substrate (Thermo Fisher Scientific, Rockford, IL, US, catalog no. 34095) or Immobilon Western Chemiluminescent HRP substrate (Millipore, Burlington, MA, US, catalog no. WBKLS0500). Signal intensity was measured using ImageJ 64 software.

### Cell cycle

1.2×10^5^ PC3 or 1.4×10^5^ DU145 cells per well were seeded in 12-well plates. After overnight culture, 100-pmol siNC or siASH1L were transfected with 4 μl of TransIntro EL Transfection Reagent. Cells were collected at 24, 48, and 72 h and washed twice with PBS before the fixation in 75% ethyl alcohol at 4°C for 40 min. After washing with PBS twice, the fixed cells were resuspended with 100 μl of PBS containing RNase A (TransGen Biotech, catalog no. GE101) with a final concentration of 20 μg/ml and incubated at 37°C for 40 min. Next, 2 μl of PI (1 mg/ml) was added per sample and stained in the dark for 15 min. The samples were kept at 4°C and analyzed using BD LSRFortessa (BD Biosciences, San Jose, CA, US). The cell cycling distributions were determined using ModFit LT software (Verity Software House, Topsham, ME, US). All experiments above were repeated with at least three trials.

### Cell apoptosis

ASH1L was knocked down in PC3 or DU145 cells with siRNA in the same way as described for cell cycle studies. Per sample was collected at 24, 48, or 72 h, washed with cold PBS buffer and stained with the TransDetect Annexin V-EGFP/PI Cell Apoptosis Detection Kit (TransGen Biotech, Beijing, China, catalog no. FA111) according to the manufacturer’s instruction. Samples were analyzed using BD LSRFortessa (BD Biosciences) in the cell cycle study. All experiments were repeated at least three times. The data were analyzed with FlowJo v.10 software. Early apoptosis was designated as Annexin V^+^/PI^-^, and late apoptosis was designated as Annexin V^+^/PI^+^. The total apoptosis contains both the early apoptosis and late apoptosis.

### Colony formation assays

At 24 or 48 h post-transfection, the PC3 or DU145 cells treated with siNC or siASH1L#1 were seeded in 6-well plates with different cell numbers (1,000, 2,000, or 3000) per well. Cells were collected after 9 (PC3) or 7 days (DU145) and were fixed with 4% PFA for 30 min and stained with 0.1% crystal violet for 30 min. Then, the plates were washed and photographed. The results were obtained from at least three independent replicates.

### MTT assay

Cells were seeded into a 96-well plate. After overnight culture, the cells from each well were transfected with 10 pmol siNC or siASH1L with 0.4 μl of TransIntro EL Transfection Reagent. After incubation for 72 h, 20 μl of the MTT (3- [4,5-dimethylthiazol-2-yl]-2,5 diphenyl tetrazolium bromide) solution was added to each well (final MTT concentration, 0.5 mg/ml), and then, the cells were incubated for 4 h in a cell incubator. Subsequently, the medium was discarded and 150 μl of DMSO were added into each well to dissolve the formazan crystals, and the absorbance of each well was measured at a wavelength of 490 nm by MULTISKAN GO (Thermo Scientific, Waltham, MA, US). All experiments were repeated at least three times.

### RNA isolation, reverse transcription, and quantitative Real-Time Polymerase Chain Reaction

RNA was extracted with the RNAiso Plus reagent (Takara Bio, Shiga, Japan, catalog no. 9109) and reverse-transcribed with Hifair III 1st Strand cDNA Synthesis SuperMix for qRT-PCR (Yeasen Biotechnology, Shanghai, China, catalog no. 11141ES60). The reverse transcription products of different samples were amplified by a LightCycler System (Roche, Mannheim, Germany) using the SYBR Green Master Mix (Yeasen Biotechnology, Shanghai, China, catalog no. 11201ES08) according to the manufacturer’s instruction, and data were normalized by the level of GAPDH in each sample. The 2^-ΔΔCt^ method was used to calculate relative expression changes. With the help of the dissociation curve analysis, specific primer pairs of every gene were designed and selected. The sequences of the primers for qRT-PCR are shown in **Supplemental Table S1**. All experiments were repeated at least three times.

### RNA-Seq analysis

PC3 cells were plated at a 6-well plate with 3.5 × 10^5^ cells per well. After overnight culture, 250- pmol siNC or siASH1L#1 were transfected with 10 μl of the EL transfection reagent (TransGen Biotech, catalog no. FT201) per well as dictated by the protocol. The cells were collected at 48 h; 10% of the cells were treated with the RNAiso Plus reagent, and the remaining cells were quickly frozen with liquid nitrogen. The knockdown efficiency of the ASH1L gene was confirmed by qRT-PCR after mRNA extraction. The frozen samples were mailed to BGI Bioinformatics Corporation (Wuhan, China) with the project number: F21FTSNCKF3033_HONgbclN. The RNA preparation, library construction, and sequencing using the DNBSEQ platform were performed at BGI. RNA sample quality was examined by Bioanalyzer (Agilent 2100). After getting clean reads, Bowtie2 (version: v2.2.5) was used to map the clean reads to the reference gene sequence (transcriptome), and the gene expression level of each sample was calculated with RSEM (v1.2.8). The DEseq2 method was used for differential gene detection, and according to results of the differential gene detection, the R Package pheatmap was used to perform hierarchical analysis on the union set differential genes. The data were analyzed using the BGI Dr. Tom multi-omics integration analysis website (https://biosys.bgi.com/#/report/login).

### Statistical analysis

All statistical analyses were performed using GraphPad Prism 7 (GraphPad Software). Statistical significance was analyzed using a two-tailed unpaired Student’s *t*-test for the comparison of two conditions. *P* < 0.05 was considered statistically significant. Statistical significance levels were denoted as follows: *P < 0.05; **P < 0.01; ***P < 0.001. Experimental data are presented as mean ± SEM unless stated otherwise. Sample numbers are indicated in the relevant figure legends.

## Results

### ASH1L–plant homeodomain finger recognizes three methylated states of H3K4 with comparable affinities

To determine whether the ASH1L PHD finger has binding preference for H3K4 methylation states, we investigated the binding selectivity of the ASH1L PHD finger using a histone peptide microarray, an isothermal titration calorimetry (ITC) assay, and a nuclear magnetic resonance (NMR) titration assay. In the histone peptide microarray analysis, ASH1L PHD was able to bind H3K4me1, H3K4me2, and H3K4me3 peptides with equivalent intensities (**Figure 1B**) but did not interact with the unmethylated H3K4 peptide (data not shown). The ITC assay showed that ASH1L PHD was bound with comparable affinities to H3K4me1 (K_D_ ~ 75 μM), H3K4me2 (K_D_ ~ 39 μM), and H3K4me3 (K_D_ ~ 64 μM) peptides but showed only marginal binding to the unmodified H3K4 peptide (K_D_ > 300 μM) (**Figures 1C, D**). Furthermore, we carried out ^1^H,^15^N heteronuclear single quantum coherence (HSQC) NMR titration experiments. H3 (aa 1-15) peptides containing different K4 methylation states in a concentration range between 25 and 2,000 μM were respectively titrated into the ^15^N-labeled ASH1L PHD protein solution, and the ^1^H,^15^N HSQC were recorded and compared to the ASH1L PHD spectrum without adding the H3 peptide. Chemical shift perturbations in the intermediate exchange regime on the NMR time scale displayed that the addition of H3K4me1, H3K4me2, or H3K4me3 peptide (protein-to-peptide molar ratio, 1:5) produced similar perturbation patterns (**Figure 1E**). However, the addition of the non-methylated H3K4me0 peptide did not induce perceivable chemical shift changes (**Supplemental Figure S1A**). This indicates that the PHD domain undertakes similar structural conformation changes after interacting with three different H3K4 methylation peptides rather than the unmethylated peptide. The K_D_ values from NMR titration experiments revealed that the ASH1L PHD domain was bound with comparable affinities to H3K4me1 (K_D_ ~ 181 μM), H3K4me2 (K_D_ ~ 238 μM), and H3K4me3 (K_D_ ~ 106 μM), although its binding to the H3K4me3 peptide was slightly stronger than bindings to H3K4me1 and H3K4me2 peptides. (**Figures 1C, F**, and **Supplemental Figure S1B**). In addition, we evaluated ASH1L PHD binding with other H3-derived methylated peptides (H3K9me2 and H3K36me2) by using NMR HSQC and ITC experiments and found that the PHD domain had weak binding affinity for both H3K9me2 and H3K36me2 peptides (ITC, K_D_ > 500 μM) (**Supplemental Figures S1C, S1D**). Together, these results show that the ASH1L PHD finger is a specific binding module for the N-terminal tail of histone H3 with a different lysine methylation state at H3K4.

In ASH1L, an atypical bromodomain is in tandem with the PHD finger, but its role in ASH1L histone methyltransferase activity remains largely unknown. To examine whether ASH1L bromodomain facilitates the recognition of methylated H3K4, we expressed and purified the Bromo-PHD tandem domain (residue 2425–2627) and measured its binding affinity against different methylated H4K4 peptides. ITC analysis showed that the Bromo-PHD tandem domain bound H3K4me1 (K_D_ ~ 64 μM), H3K4me2 (K_D_ ~ 29 μM), and H3K4me3 (K_D_ ~ 48 μM) peptides with comparable affinities but had weak binding to the unmodified H3K4 peptide (K_D_ > 500 μM) (**Supplemental Figure S2A, S2B**). These results correlated with the K_D_ values of the PHD-domain-alone binding to H3K4 peptides with different methylation states (**Figures 1C, D**), suggesting that the ASH1L bromodomain did not enhance the binding ability of the Bromo-PHD tandem domain to the N-terminal methylated histone H3K4.

### Structure of ASH1L plant homeodomain finger binding with H3K4me2 (1–15) peptide

**Figure 2:**
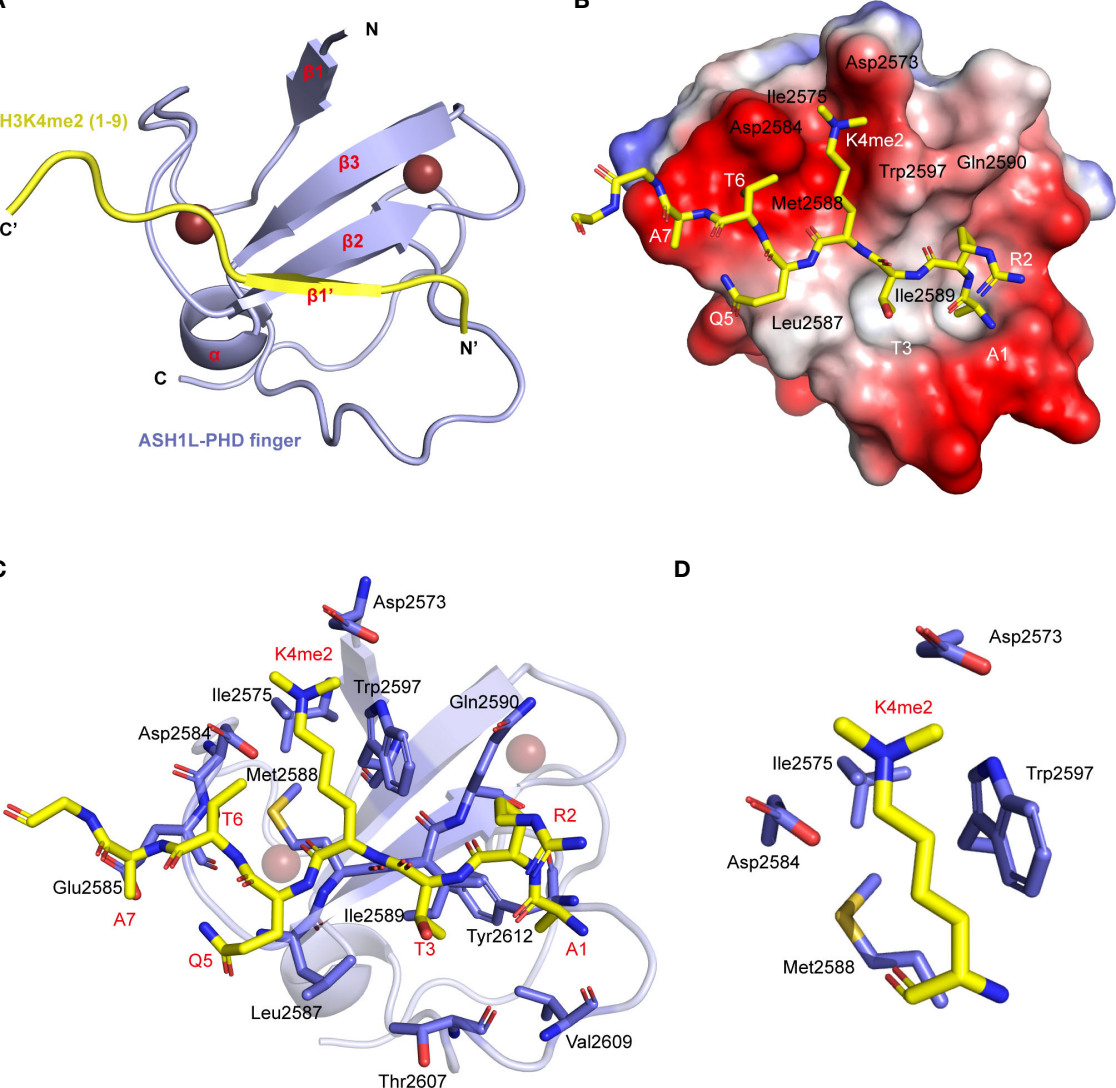
Structure of ASH1L PHD finger binding with the H3 (1–15) K4me2 peptide. **(A)** Cartoon representation of the NMR structure of the ASH1L PHD finger (light blue)/H3K4me2 peptide (yellow). **(B)** Electrostatic potential surface representation of the ASH1L PHD finger binding with H3K4me2 peptide in stick depiction (color-coded by atom type). The amino acid around the K4 residue and in the β2 strand are labeled. **(C)** The key residues involved in the intermolecular interactions at the protein/peptide interface are labeled. These side chains are color-coded by the atom type and showed in the stick model. **(D)** The key side chains of binding groove around K4me2 are picked out.

To understand the molecular basis for recognizing different states of H3K4 methylation peptides, we solved the 3D structures of the ASH1L PHD finger (residue 2573–2624 of mASH1L) in complex with the H3K4me2 peptide (residue 1–15) (**Figure 2**) using triple-resonance NMR spectroscopy methods (54). The final Root Mean Squared Distance (RMSD) of 0.22 ± 0.029 A for the backbone atoms of the PHD domain and the five N-terminal residues of the H3 peptide indicates that these structures are well defined (**Supplemental Table S2**). The ASH1L PHD finger consists of a double-stranded antiparallel β-sheet (β2, β3) and a helical turn (α1), which are anchored by two zinc atoms in a cross-brace topology fold. In addition to the canonical PHD-like fold (51), a short the β-sheet (β1) is also present at the N-terminus of the protein. The H3 peptide adopts an extended conformation and forms an antiparallel β-strand (β1’) to the Leu2587-Gln2590 segment of the protein (**Figures 2A, B**). The seven N-terminus residues of the H3K4me2 peptide are in direct contact with the PHD, while the rest of the tail is unstructured and completely exposed to the solvent (**Figure 2B**). The amino terminus of the H3 peptide is anchored through intermolecular hydrogen bonds with the backbone carbonyl oxygen atoms of protein residues Thr2607 and Val2609 and non-polar interactions between the methyl group of H3A1 and the side chains of Ile2589, Val2609, and Tyr2612 (**Figure 2C**). The peptide is also stapled to the protein surface with non-polar interactions formed between the side-chain groups of peptide and protein residues, that is, H3R2 to Gln2590 and Trp2597, H3T3 to Leu2587, Ile2589, Trp2597 and Thr2607, H3K4me2 to Trp2597, and H3Q5 to Leu2587, H3T6 to Asp2584 and Glu2585, H3A7 to Glu2585, respectively (**Figure 2C**). K4me2 side-chain atoms are partially caged in a narrow groove with the side-chain atoms of protein residues Asp2573, Ile2575, Asp2584, Met2588, and Trp2597 forming the rim, which provides an electronegative environment and allows for hydrophobic and cation-π interactions for the dimethyl-ammonium moiety (**Figures 2B, D**).

### Molecular basis of ASH1L plant homeodomain finger for non-selective recognition of methylated H3K4

**Figure 3:**
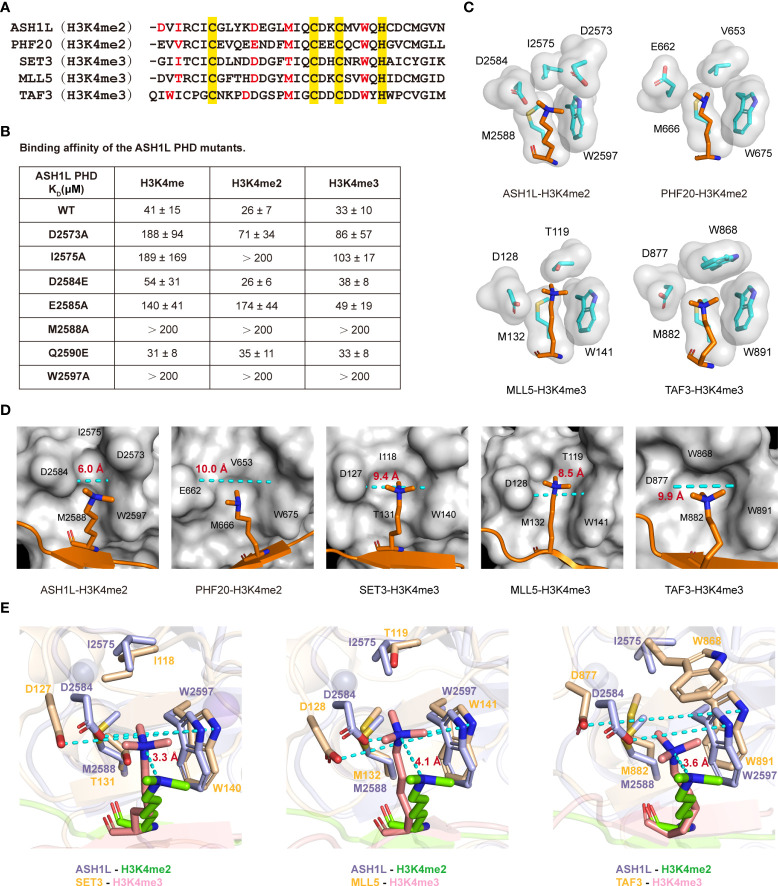
Molecular basis of the ASH1L PHD finger for non-selective recognition of methylated H3K4. **(A)** Alignment of amino acid sequences of different PHD fingers. The H3K4me2 binding site residues of ASH1L and PHF20, as well as the H3K4me3 binding site residues of SET3, MLL5, and TAF3, are colored red. The conservative skeleton amino acids are colored yellow. The following PDB structures were used in this figure: 5tbn (PHF20), 4l58 (MLL5), 5tdw (SET3), and 2k17 (TAF3). **(B)** Binding affinities of the mutated ASH1L PHD finger with the indicated histone peptides are measured by ITC. **(C)** The binding pockets of the different PHD fingers are illustrated by showing the side chains (cyan stick and surface representation) that surround the methylated H3K4 ligand residue (orange stick). **(D)** Surface plots of ASH1L, PHF20, SET3, MLL5, and TAF3 PHD finger binding with H3K4me2 or H3K4me3 peptide (orange). The side chain of K4 is in a stick mode, and the other part of H3 peptide is in a cartoon mode. The distance between the aspartic side chain and the tryptophan aromatic ring are measured using Pymol software and labeled. **(E)** Superimposed H3K4me2 (chartreuse) binding site of ASH1L PHD finger (light blue) and H3K4me3 (salmon) binding sites of SET3, MLL5, and TAF3 PHD fingers (wheat) are shown. The distance between the methyl-ammonium moiety of K4 in different PHD finger structures are measured using Pymol software and labeled.

To elucidate how the ASH1L PHD finger can bind all three methylation states of H3K4, we aligned amino acid sequences of the PHD fingers from MLL5 (H3K4me3) (45), TAF3 (H3K4me3) (64), SET3 (H3K4me3) (48), and PHF20 (H3K4me2) (49) with the ASH1L PHD finger (**Figure 3A**). Two residues in the methylated-lysine binding pocket are ultimately conserved: a methionine and a tryptophan (Met2588 and Trp2597 in ASH1L PHD, colored red, **Figure 3A**). The mutation of Met2588 or Trp2597 to an alanine completely abolished the interaction of ASH1L PHD to all three methylation states of H3K4 peptides (**Figure 3B**). The mutation of Ile2575 to Ala also significantly weakened the binding, because the smaller alanine residue reduces the hydrophobic interaction between the protein and peptide. The Gln2590 mutation to a glutamate did not cause considerable changes in binding to all three methylation states, indicating that the residue is not likely to be involved in stabilizing the peptide interaction (**Figure 3B**). Collectively, these results validated that conserved Ile2575, Met2588, and Trp2597 residues in the methylated-lysine binding pocket are necessary for the protein-and-peptide interaction.

We next examined methylated lysine binding pockets in the structures of MLL5, TAF3, SET3, and PHF20 PHD fingers. The binding pockets of PHD fingers that recognize H3K4me3 peptides (MLL5, TAF3) contain a negatively charged aspartate residue (Asp128 in MLL5, Asp877 in TAF3), which contributes to the specific H3K4me3 binding (**Figure 3C**) (45, 64). However, in the PHF20 PHD–binding pocket, a longer glutamate residue (Glu662) is important for the H3K4me2 binding (**Figure 3C**) (49). The mutation of Glu662 to Asp significantly increases the binding affinity of PHF20 PHD to the H3K4me3 peptide (49). These findings demonstrate that the shorter aspartic side chain in PHD fingers may accommodate the pocket space to selectively bind the larger trimethylation states of H3K4, whereas the longer glutamic side chain adapts the pocket space for the smaller dimethylation state of H3K4. Intriguingly, the ASH1L PHD finger had an aspartate residue (Asp2584) in the corresponding binding rim but did not show binding discrimination against the three methylated-lysine states of H3K4 peptides (**Figures 3B, C**). Furthermore, the mutation of Asp2584 to Glu did not alter the binding preference to any three methylation H3K4 peptides, exhibiting similar affinities for H3K4me (K_D_ ~ 54 μM), H3K4me2 (K_D_ ~ 26 μM), and H3K4me3 (K_D_ ~ 38 μM) (**Figure 3B**). However, the mutation of the subsequent residue Glu2585 to Ala decreased the PHD binding to H3K4me and H3K4me2 peptides by ~3-fold and ~7-fold, respectively, but had no perceivable effect on the binding affinity of H3K4me3 (**Figure 3B**). These results suggest that Asp2584 in ASH1L PHD affects the binding selectivity to a less extent than the conserved aspartate residues in MLL5 and TAF3 PHD fingers, whereas Glu2585 may be required for proper conformational folding to the ASH1L PHD structure.

To further characterize the binding specificity of the conserved aspartate residue in ASH1L, we compared the K4me2-binding groove of ASH1L and PHF20 PHD with the K4me3-binding pocket of MLL5, TAF3, and SET3 PHD, and evaluated the solvent-accessible pocket surface (**Figure 3D**). We found that the K4me2-binding groove in ASH1L was relatively narrower than the K4me2-binding pocket in the PHF20 PHD finger and K4me3-binding pockets in MLL5, TAF3, and SET3 PHD fingers. Particularly, the distance between the conserved aspartic side chain and the tryptophan aromatic ring for ASH1L PHD was approximately ~6.0 Å. This is much shorter than the distances between the equivalent Asp and Trp residues in SET3 (~9.4 Å), MLL5 (~8.5 Å), and TAF3 (~9.9 Å) PHD domains or between Glu662 and Trp675 in the PHF20 PHD–binding pocket (~10.0 Å) (**Figure 3D**). Consequently, the dimethyl-ammonium moiety of K4 in the ASH1L complex was excluded from the binding core and exposed toward the solvent, and its relative position shifted outward ~3.3 Å in comparison to that of the trimethyl-ammonium moieties in SET3, ~4.1 Å in MLL5 and ~3.6 Å in TAF3 complexes (**Figure 3E**). Notably, the side chain of Asp2573 at the N-terminal loop swings toward the core of the ASH1L PHD finger (**Figure 3C**), and the mutation of Asp2573 to Ala significantly weakened the binding between ASH1L PHD and all three methylation states of H3K4 (**Figure 3B**). This suggests that the side chain at this structural position likely forms an electrostatic contact with the positively charged dimethyl-ammonium moiety of the H3K4me2 peptide, thereby stabilizing the complex. Together, these structural and biochemical analyses demonstrate that the narrow binding groove and unique Asp2573 residue present in the K4me2-binding pocket are major determinants of the non-methylation state specificity and weak binding affinities of the ASH1L PHD finger (**Figure 3E**, **Supplemental Table S3**).

### ASH1L is required for gene expression regulation in cell cycle and apoptosis

**Figure 4:**
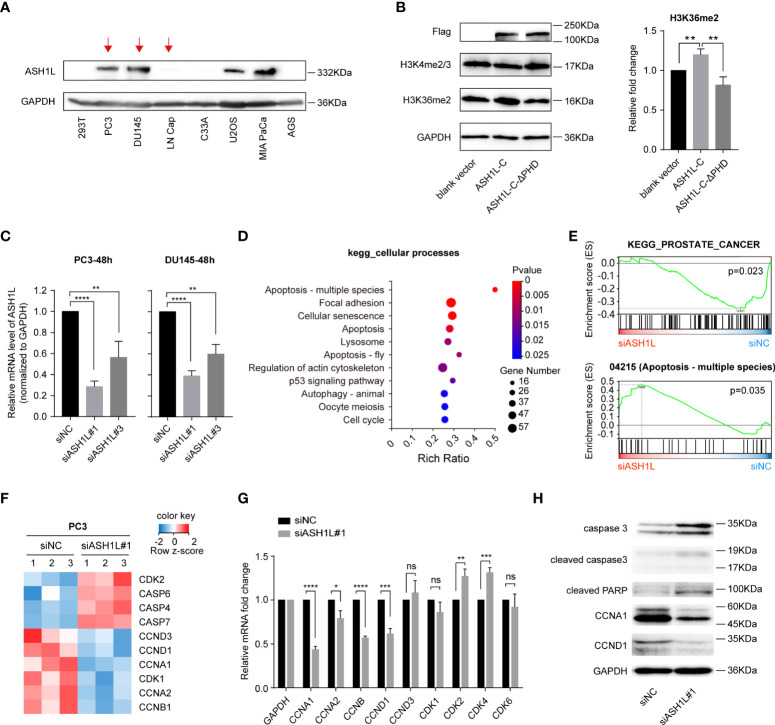
ASH1L is required for gene expression regulation in the cell cycle and apoptosis. **(A)** Western blot analysis of ASH1L protein levels in different cancer cell lines. The GAPDH was used as a loading control. **(B)** Western blot analysis of H3K4me2/3 and H3K36me2 levels after Flag-ASH1L-C or Flag-ASH1L-C-ΔPHD overexpressed in 293T cells. **(C)** The knockdown efficiency of siRNAs targeting ASH1L (siASH1L) was detected by qRT-PCR at 48 h after siNC and siASH1L were transfected to PC3 or DU145 prostate cancer cell lines. **(D)** The analysis of differentially expressed genes (DEGs) in the cellular processes branch of the KEGG pathway. The RNA-seq result is from the three replicates of the control (siNC) group and ASH1L knockdown (siASH1L#1) group PC3 cells. **(E)** GSEA of RNA-seq data to analyze the signaling pathways altered by ASH1L knockdown in PC3 cells. **(F)** Heatmap showing changes in the expression of indicated genes in the control (siNC) and ASH1L knockdown (siASH1L#1) PC3 cells. **(G)** qRT-PCR analysis of cell cycle–related gene expression in the control (siNC) and ASH1L knockdown (siASH1L#1) PC3 cells. **(H)** Western blot analysis of cell cycle and apoptosis pathway–related proteins in the control (siNC) and ASH1L knockdown (siASH1L#1) PC3 cells. The GAPDH was used as loading control. The density of every band was quantified using Image J software. (These experiments were repeated at least three times, and the error bars represent at least three replicates. ns, not significant, *p < 0.05, **p < 0.01, ***p < 0.001, ****p < 0.0001).

The dysregulation of ASH1L is known to promote the progression of various aggressive solid cancers (36–40) and is required for the activation of tumor-related gene expression and cellular growth (34, 37). To explore the biological function of ASH1L in cancers, we evaluated the ASH1L protein levels in different cancer cell lines, such as androgen-sensitive PCa LNCaP, androgen receptor (AR)–negative CRPC (PC3, DU145), human cervical carcinoma cell line C33A, human bone osteosarcoma epithelial cell line U2OS, human pancreatic carcinoma cell line MIA-PaCa2, human gastric cancer cell line AGS, and human embryonic kidney cell line 293T. Western blot analysis revealed a significant increase of ASH1L protein levels in PC3 or DU145 in comparison to LNCaP cells (**Figure 4A**), indicating that the upregulation of ASH1L likely contributes to the transition from androgen-sensitive PCa to AR-negative CRPC. ASH1L protein levels were also elevated in U2OS and MIA-PaCa2 cells but remained relatively low in C33A, AGS, and 293T cells (**Figure 4A**), implying its tissue-specific role in different cell types.

To investigate whether the PHD domain facilitates ASH1L-mediated histone methylation in cells, we transfected either the C-terminal active core of ASH1L (Flag-ASH1L-C, aa 1892-2969) or the PHD deletion mutant Flag-ASH1L-C-ΔPHD into 293T cells (**Figure 1A**, **Supplemental Figure S3A**) (32). Western blot analysis showed that the basal level of ASH1L was low in 293T cells in comparison to AR-negative PC3 and DU145 cells (**Figure 4A**). After the overexpression of Flag-ASH1L-C, the overall level of H3K36me2 rather than H3K4me2/3 increased (**Figure 4B**), indicating that Flag-ASH1L-C promoted the dimethylation of histone H3K36 but not H3K4. However, the overexpression of the deletion mutant Flag-ASH1L-C-ΔPHD only showed marginal modulation to either H3K36me2 or H3K4me2/3 protein levels (**Figure 4B**), suggesting that the deletion of the PHD domain likely abolished the recruitment of ASH1L-C-ΔPHD to chromatin and consequently suppressed the ASH1L-dependent H3K36 methylation.

To examine whether ASH1L has an oncogenic role in CRPC development, we then knocked down the ASH1L gene in PC3 and DU145 cells using ASH1L-specific siRNAs and investigated the molecular functions of ASH1L in CRPC cells. ASH1L silencing efficiency in the transfected cells was assessed using qRT-PCR analysis, and the data showed ~70% (or ~60%) inhibition of ASH1L mRNA expression in PC3 (or DU145) cells after 48 h of transfection with siASH1L#1 relative to its expression in cells transfected with siNC (**Figure 4C**). We next conducted RNA sequencing (RNA-seq) analysis from PC3 cells with siRNA-mediated ASH1L knockdown. Kyoto Encyclopedia of Genes and Genomes (KEGG) pathway enrichment analysis demonstrated that upon ASH1L knockdown, targeted genes were significantly enriched in cancer-related pathways, such as apoptosis, focal adhesion, cellular senescence, and cell cycle (**Figure 4D**). The gene set enrichment analysis (GSEA) of RNA-seq results indicated the altered genes by ASH1L knockdown were mainly associated with gene sets linked to the PCa, apoptosis, and cell cycle signaling cascades (**Figure 4E** and **Supplemental Figure S3B**). The regulators of apoptosis, including *CASP4, CASP6*, and *CASP7* (65, 66), and the regulators of CDK kinases in cell cycle progression, including *CCNA1, CCNA2, CCNB1, CCND1*, and *CCND3* (67, 68) were particularly altered by ASH1L knockdown (**Figure 4F**). We then performed qRT-PCR analysis to assess the mRNA transcript levels of cell cycle–regulator genes *CCNA1*, *CCNA2*, *CCNB*, *CCND1*, and *CCND3* and cyclin-dependent-kinase genes *CDK1*, *CDK2*, *CDK4*, and *CDK6*. We found that *CCNA1*, *CCNA2*, *CCNB*, and *CCND1* mRNA levels were significantly reduced, following ASH1L knockdown, whereas *CDK2* and *CDK4* levels increased slightly (**Figure 4G**). In addition, Western blot analysis showed that the knockdown of ASH1L resulted in a marked increase in the proteolytic cleavage of PARP, a signature consequence during apoptosis (**Figure 4H**). ASH1L knockdown also elevated the levels of caspase-3 and the cleavage of caspase-3 (**Figure 4H**), an upstream activator of PARP cleavage. The downregulation of CCNA1 and CCND1 after ASH1L knockdown was also observed by Western blot (**Figure 4H**). Collectively, these results validated the critical role of ASH1L in regulating the cell cycle and apoptosis in PC3 cells.

In addition, we analyzed available ChIP-seq data (GSE147074) with or without ASH1L knockout in anaplastic thyroid carcinoma BHT-101 cells and found that ASH1L knockout significantly reduced the H3K36me2 enrichment at some oncogene loci including *APLP2*, *SERPINE1*, *NEK7*, *LAMB3*, and *ITGA6* (**Supplemental Figure S3C**) and consequently modulated the expressions of these genes (37). These results were in agreement with our RNA-seq analysis that the mRNA levels of the same set of genes were substantially decreased after the ASH1L knockdown in PC3 cells. Notably, these genes play important roles in the growth, apoptosis, and migration of tumor cells. Our qRT-PCR analysis further confirmed that ASH1L knockdown suppressed the expression of these key oncogenes in PC3 cells (**Supplemental Figure S3D**), demonstrating that ASH1L-dependent H3K36 methylation is important for maintaining active gene expression.

### ASH1L knockdown induced cell cycle arrest and apoptosis but did not affect cell viability

**Figure 5:**
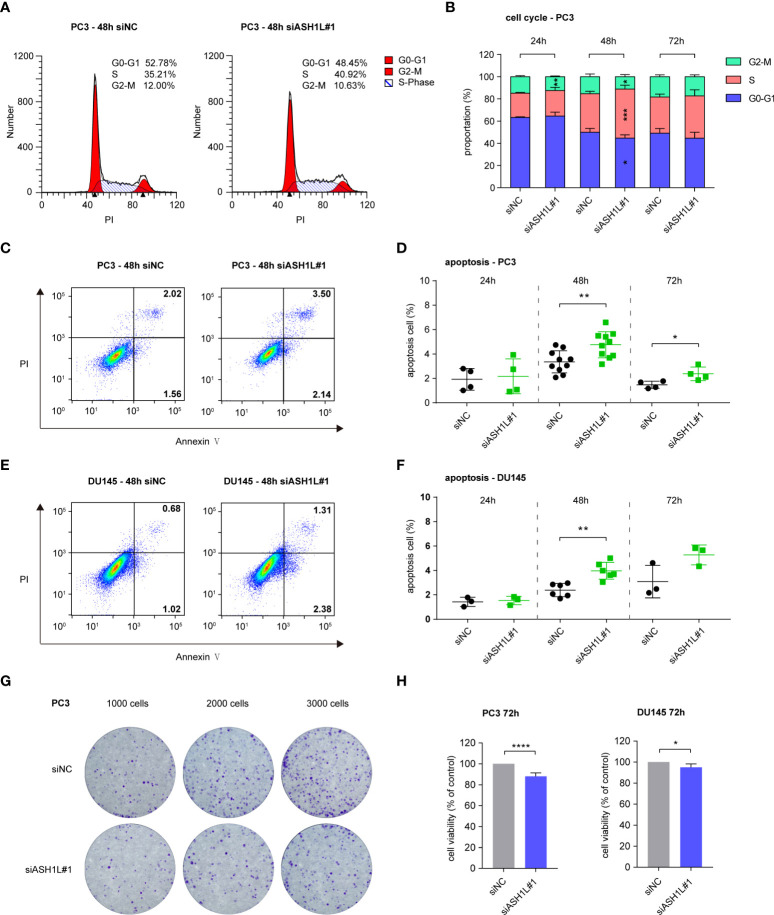
ASH1L knockdown induced cell cycle arrest and apoptosis but did not affect cell viability. **(A)** Representative flow cytometry images about the cell cycle distribution of PC3 at 48 h after siNC and siASH1L#1 transfection. **(B)** Cell cycle analysis of PC3 at 24, 48, and 72 h after siNC and siASH1L#1 transfection. **(C)** Representative flow cytometry images showing the proportion of early apoptotic (Annexin V^+^ PI^-^) and late apoptotic (Annexin V^+^ PI^+^) PC3 cells at 48 h after siNC and siASH1L#1 transfection. **(D)** Apoptosis level of PC3 detected by Annexin V/PI staining at 24, 48, and 72 h after siNC and siASH1L#1 transfection. **(E)** Representative flow cytometry images showing the proportion of early apoptotic (Annexin V^+^ PI^-^) and late apoptotic (Annexin V^+^ PI^+^) DU145 cells at 48 h after siNC and siASH1L#1 transfection. **(F)** Apoptosis level of DU145 detected by Annexin V/PI staining at 24, 48, and 72 h after siNC and siASH1L#1 transfection. **(G)** Plate colony formation was performed in PC3 cells transfected with siNC and siASH1L#1 and incubated for 9 days. Representative colony images are shown. **(H)** Analysis of cell viability by the MTT assay in siNC- and siASH1L#1-transfected PC3 cells. The plates were detected at 72 h after siNC and siASH1L#1 were transfected. (These experiments were repeated at least three times, and the error bars represent at least three replicates. *, p < 0.05, **, p < 0.01, ****, p < 0.0001).

We next studied the cell cycle distribution by flow cytometry in PC3 and DU145 cells. After cells were transfected with siRNA for 48 h, we found that the knockdown of ASH1L induced S arrest and decreased the fractions of G0-G1 and G2-M phases in both PC3 and DU145 cells. Notably, the ASH1L knockdown induced more S arrest and decreased the fractions of the G0-G1 phase and G2-M phases in PC3 cells than those in DU145 cells (**Figures 5A, B** and **Supplemental Figure S4**). This suggests that ASH1L is more involved in cell cycle regulation in PC3 than in DU145 cells. Furthermore, depleting ASH1L may facilitate cell proliferation inhibition, at least in part by inducing cell cycle arrest at the S phase. We also used flow cytometry to determine whether the reduced expression of ASH1L might be associated with apoptosis. The depletion of ASH1L increased the percentage of apoptotic cells in PC3 and DU145 cells compared with the siNC groups at 48 h (4.8 ± 0.34% vs. 3.4 ± 0.29% for PC3 and 4.0 ± 0.28% vs. 2.4 ± 0.22% for DU145) (p<0.01) (**Figures 5C–F**), indicating that the ASH1L is involved in CRPC cell survival. We then investigated the effect of ASH1L knockdown on colony formation in PC3 cells and observed lower colony numbers, following the ASH1L knockdown, suggesting that depleting ASH1L could inhibit colony formation in PC3 cells (**Figure 5G**). Intriguingly, the MTT assay data showed that the knockdown of ASH1L moderately decreased cell viability in both PC3 and DU145 cell lines (**Figure 5H**). Collectively, these results indicate that decreasing ASH1L expression exerts an antitumor effect by inducing S arrest and apoptosis and inhibiting colony formation in PC3 and DU145 cells.

## Discussion

In this study, we demonstrate that the ASH1L PHD finger non-selectively binds all three states of methylated-lysine H3K4 to a similar extent but only discriminates against the unmethylated H3K4me0. This unique selectivity of the ASH1L PHD finger differs from that of other PHD fingers, which generally prefer to interact with either H3K4me2 or H3K4me3 (**Supplemental Table S3**). Other PHD domains adopt the specific interaction through an additional electrostatic association involving the di- or trimethylammonium group of lysine and the carboxyl group of Glu or Asp and a commodious binding pocket suitable to the methylated-lysine side chain. However, the ASH1L PHD finger does not form this electrostatic contact. Instead, our structural analysis illustrates that the ASH1L PHD finger relies largely on hydrophobic and cation–π interactions to accommodate the dimethyl-ammonium moiety. Intriguingly, an electronegative aspartate residue (Asp2573) at the N-terminal of PHD plays an important role for this binding. Furthermore, the narrow groove in the binding pocket of the ASH1L PHD finger likely leads to weaker binding affinities in comparison to other PHD fingers, suggesting that ASH1L may dynamically attach and detach from chromatin in the regulation of gene transcription.

PCa is the second most common male malignancy and one of the leading causes of mortality in the world (69). Despite the significant improvements in diagnosis and systematic treatment, advanced PCa often progresses to incurable metastatic CRPC (70). Previous reports indicate that the histone methylases and demethylases can participate in the regulation of initiation, metastasis, and invasion of PCa (71–73). In this study, we found that ASH1L protein was highly expressed in AR-negative CRPC (PC3, DU145) cell lines in comparison to the androgen-sensitive PCa (LNCaP) cell line, indicating that the upregulation of ASH1L likely contributes to the transition from androgen-sensitive PCa to AR-negative CRPC. KEGG pathway enrichment analysis showed that targeted genes in the ASH1L knockdown were highly enriched in cellular processes including: apoptosis, focal adhesion, cellular senescence, and the cell cycle pathway. Furthermore, we found that the knockdown of ASH1L in PC3 cells induced more S arrest and decreased the fraction of the G0-G1 phase and G2-M phase than that in DU145 cells, suggesting that PC3 and DU145 likely have distinct mechanisms for CRPC tumor cell progression and PCa heterogeneity and that ASH1L may play a bigger role in the regulation of cell cycle in PC3 than in DU145 cells. Notably, the depletion of ASH1L induced cellular apoptosis in both PC3 and DU145 cells, suggesting that ASH1L could be a potential drug target for the therapeutic treatment of CRPC.

## Data availability statement

The datasets presented in this study can be found in online repositories. The NMR data is deposited in the Protein Data Bank (PDB) with a PDB ID 7Y0I and a BioMagResBank (BMRB) code 36492. The RNA-seq data is deposited in the Sequence Read Archive (SRA) with the accession number PRJNA821709.

## Author contributions

LZ and HY conceived the project. MY performed experiments. MY and YJ performed qRT-PCR experiments. MY, YJ, and CW collected and analyzed NMR data. ZM, DJ, and QZ carried out the protein expression and purification. MY and YL analyzed sequencing data. MY and LZ wrote the manuscript with input from all coauthors. All authors contributed to the article and approved the submitted version.

## Funding

This work was supported in part by the research fund from the First Hospital of Jilin University (Changchun, China), the Open Project of State Key Laboratory for Supramolecular Structure and Materials, JLU (SKLSSM201602), JLU Science and Technology Innovative Research Team (JLUSTIRT, 2017TD-25), International Center of Future Science, JLU, and National Natural Science Foundation of China (31770780; L.Z.).

## Acknowledgments

We thank the technical support of NMR facility at the First Hospital of Jilin University. We thank the State Key Laboratory of Supramolecular Structure and Materials at Jilin University for the use of their research facilities. We would like to acknowledge the BGI Group for generating RNA-seq data.

## Conflict of interest

The authors declare that the research was conducted in the absence of any commercial or financial relationships that could be construed as a potential conflict of interest.

## Publisher’s note

All claims expressed in this article are solely those of the authors and do not necessarily represent those of their affiliated organizations, or those of the publisher, the editors and the reviewers. Any product that may be evaluated in this article, or claim that may be made by its manufacturer, is not guaranteed or endorsed by the publisher.
