# Supplementary material for: Structural insight into ASH1L PHD finger recognizing methylated histone H3K4 and promoting cell growth in prostate cancer
Source: Front Oncol. 2022 Aug 10;12:906807. doi: 10.3389/fonc.2022.906807 (PMC9399681; doi:10.3389/fonc.2022.906807)
Supplement: Supplementary file 1 [file DataSheet_1.pdf]

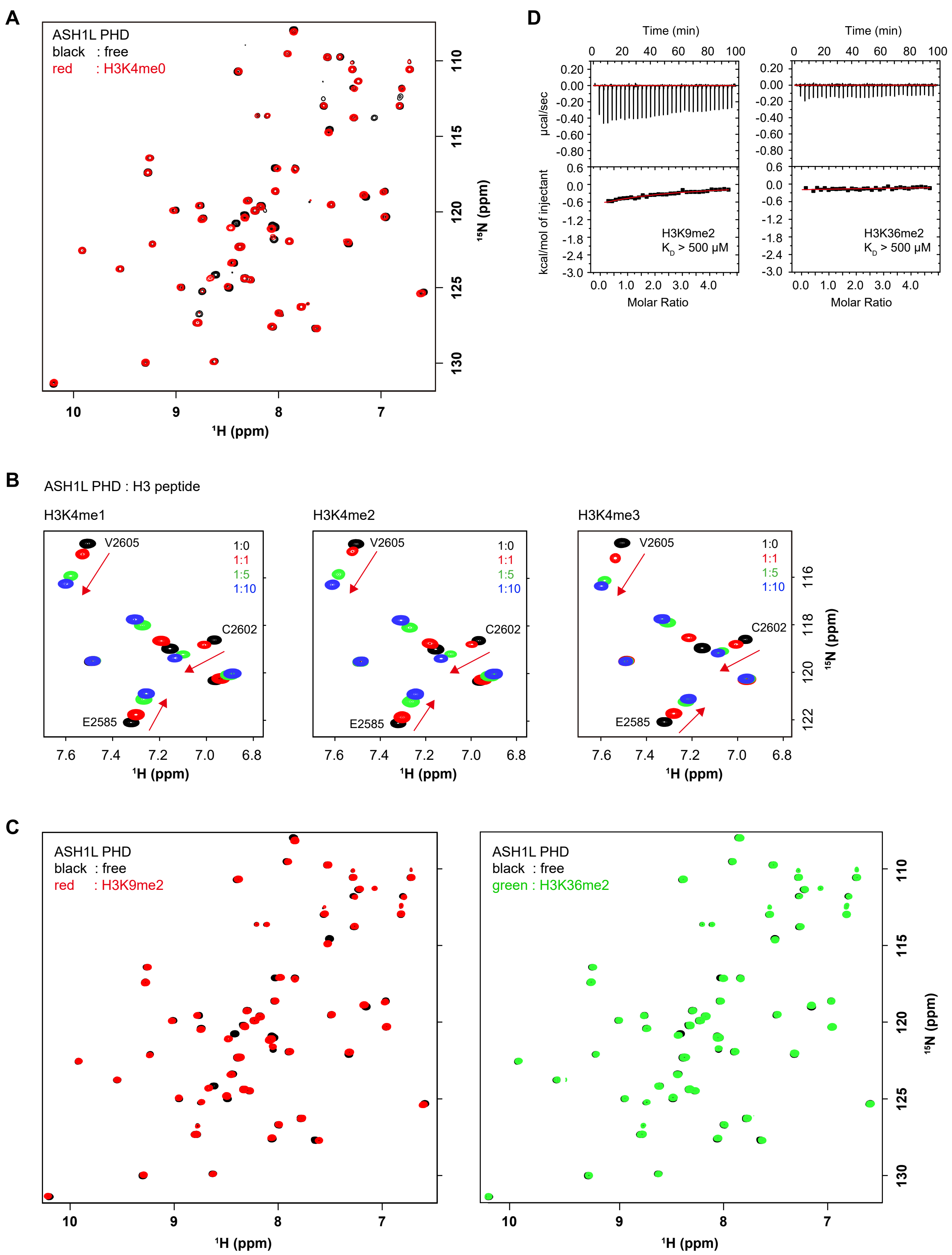

Supplemental Figure S1

ASH1L PHD finger binds with methylated H3K4 peptides but not H3K4me0, H3K9me2, or H3K36me2 peptides.

A. Superimposed ^{1}H, ^{15}N HSQC spectra of ASH1L PHD finger collected without peptide (free) or upon titration with the unlabeled H3K4me0 peptide in a protein : peptide molar ratio of 1 : 5.

B. Superimposed ^{1}H, ^{15}N HSQC spectra of ASH1L PHD finger titrated with indicated peptides. Spectra are color coded according to the ASH1L PHD: peptide molar ratio. Arrows indicate chemical shift changes.

C. Superimposed ^{1}H, ^{15}N HSQC spectra of ASH1L PHD finger collected without peptide (free) or upon titration with the H3K9me2 or H3K36me2 peptide in a protein : peptide molar ratio of 1 : 5.

D. Representative binding curves used in K_D determination of H3K9me2 or H3K36me2 peptides with PHD finger by ITC.

A

Binding affinity of the ASH1L Bromo-PHD tandem domain

Peptide H3 (1-15)	K_D (μM)
	ITC
H3K4	> 500
H3K4me1	64 ± 9
H3K4me2	29 ± 3
H3K4me3	48 ± 7

B

ASH1L Bromo-PHD tandem domain
H3 (1-15) peptides

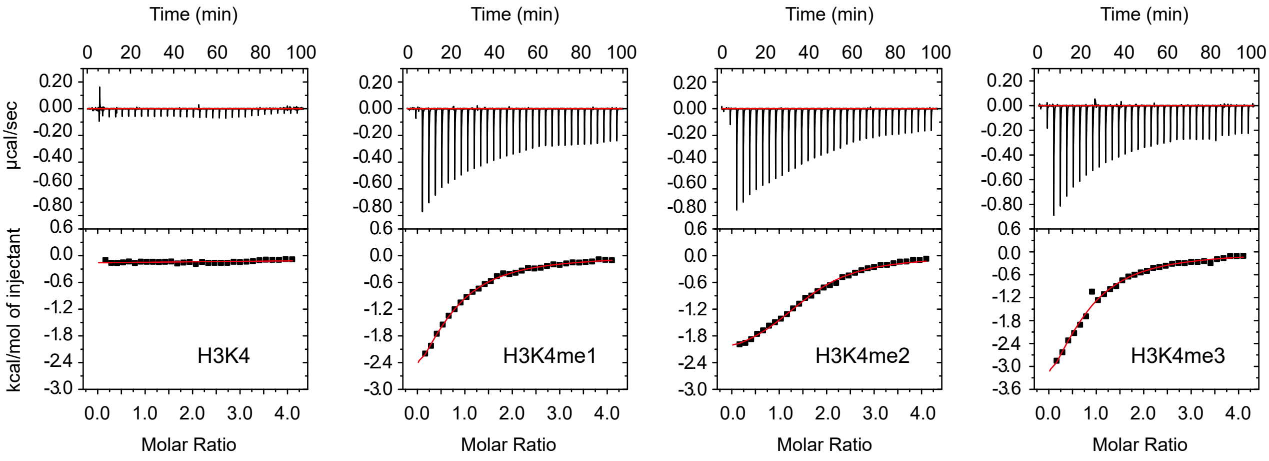

Supplemental Figure S2

The binding affinity of ASH1L Bromo-PHD tandem domain (2425-2627 aa) to different methylated H3K4 (1-15) peptides.

A. Binding affinity of ASH1L Bromo-PHD tandem domian with indicated H3 (1-15) peptides measured by ITC.

B. Representative binding carves used to determine the K_D values in ITC experiments.

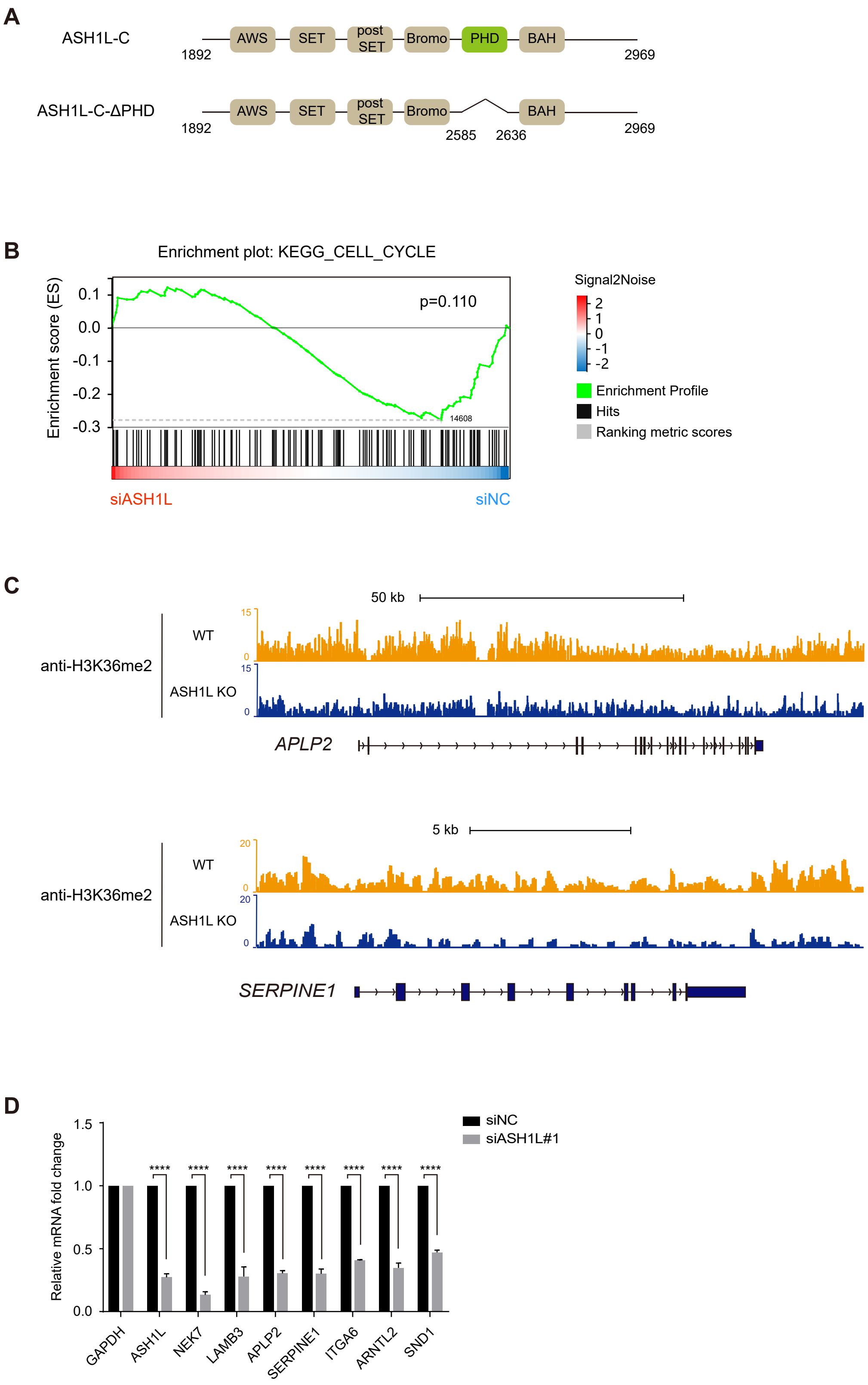

Supplemental Figure S3

ASH1L can regulate gene expression by increasing the H3K36me2 level.

A. Schematic representation of domains in Flag-ASH1L-C and Flag-ASH1L-C-ΔPHD constructs.
B. GSEA of RNA-seq data to analyze the cell cycle pathway altered by ASH1L knockdown in PC3 cells.
C. The analysis of available ChIP-seq data (GSE147074) performed in wild type (WT) and ASH1L knockout (KO) BHT-101 cells using H3K36me2 antibody. The H3K36me2 peaks on *APLP2* and *SERPINE1* are decreased after ASH1L knockout.
D. qRT-PCR verification of some downregulated genes in PC3 RNA-seq results.

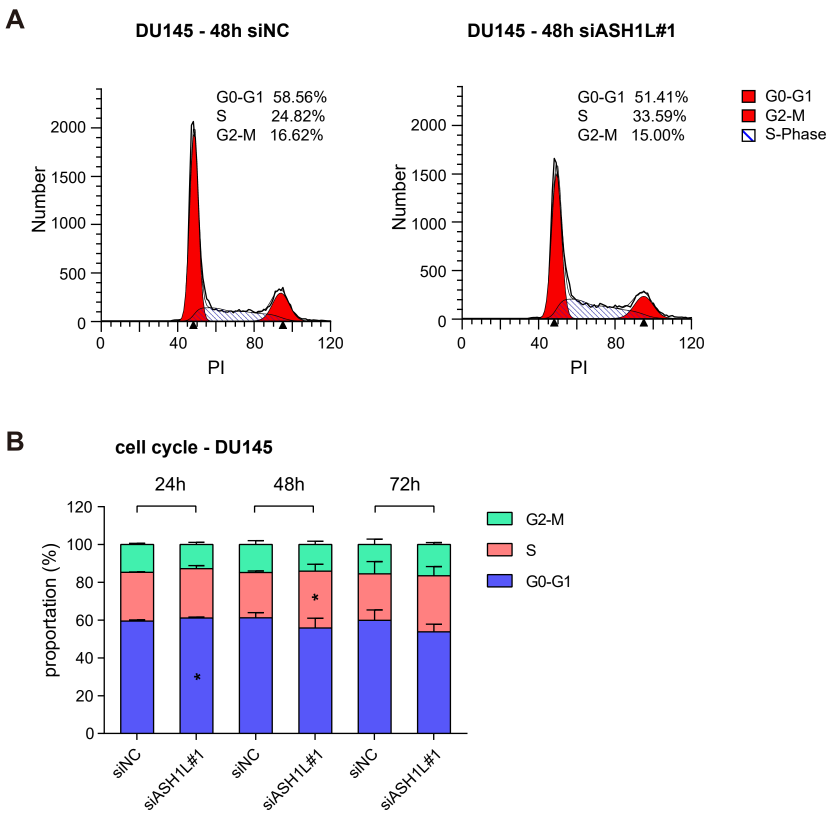

Supplemental Figure S4
ASH1L knockdown induced cell-cycle arrest in DU145.

A. Representative flow cytometry images about cell cycle distribution of DU145 at 48h after siNC and siASH1L#1 transfection.
B. Cell cycle analysis of DU145 at 24, 48, and 72h after siNC and siASH1L#1 transfection.

Supplemental Table S1

Summary of primers for qRT-PCR.

Gene	Product length	Forward primer	Reverse primer
ASH1L	75 bp	TCCTTCAAAACGAGACCCTTCA	TCTTCAGACTGGCATTCAATGG
GAPDH	130 bp	GTCTCCTCTGACTTCAACAGCG	ACCACCCTGTTGCTGTAGCCAA
CCNA1	85 bp	TAGACACCGGCACACTCAAG	AGGAGAGATGAATCTACCAGCAT
CCNA2	202 bp	GGATGGTAGTTTTGAGTCACCAC	CACGAGGATAGCTCTCATACTGT
CCNB1	228 bp	AACTTTCGCCTGAGCCTATTTT	TTGGTCTGACTGCTTGCTCTT
CCND1	195 bp	GCATCTACACCGACAACTCC	CACAGAGGGCAACGAAGGT
CCND3	173 bp	ACTGGCACTGAAGTGGACTG	ATGGCTGTGACATCTGTAGGAG
CDK1	148 bp	AAACTACAGGTCAAGTGGTAGCC	TCCTGCATAAGCACATCCTGA
CDK2	162 bp	AGCCAAGTTTCCCCAAGTG	TGGTCACATCCTGGAAGAAAG
CDK4	191 bp	GTTCGTGAGGTGGCTTTACTG	ATCGTTTCGGCTGGCAAG
CDK6	225 bp	GCTGACCAGCAGTACGAATG	GCACACATCAAACAACCTGACC

Supplemental Table S2
Summary of restraints and statistics of the final 20 structures of ASH1L PHD domain in complex H3K4me2 peptide.

	PHD/H3K4me2
Protein NMR distance and dihedral constraints	
Distance constraints	
Total NOE	1272
Intra-residue	421
Inter-residue	851
Sequential (|i - j| = 1)	232
Medium-range (2 ≤ |i - j| ≤ 5)	225
Long-range (|i - j| > 5)	394
Inter-molecular constraints	62
Hydrogen bonds	15
Total dihedral angle restraints	
Phi angle	41
Psi angle	41
Ramachandran Map Analysis (%)	
Most favored regions	94.3
Additional allowed regions	5.7
Generally allowed regions	0.0
Disallowed regions	0.0
Structure statistics	
Violations (mean +/- s.d.)	
Distance constraints (Å)	0.063 +/- 0.017
Dihedral angle constraints (º)	0.45 +/- 0.10
Max. dihedral angle violation (º)	0.62
Max. distance constraint violation (Å)	0.092
Deviations from idealized geometry	
Bond lengths (Å)	0.0053 +/- 0.00017
Bond angles (º)	0.70 +/- 0.033
Impropers (º)	1.6 +/- 0.090
Average pairwise r.m.s. Deviation ** (Å)	
Heavy	0.73 +/- 0.062
Backbone	0.25 +/- 0.047

**: The residue number ranges used in protein/peptide complex RMSD calculations are 5-52 and 301-305. Pairwise r.m.s. deviation was calculated among 20 refined structures of total 200 structures.
%: Procheck residue numbers are 6-7, 18-24, 26-31, 32-38 and 47-49.

Supplemental Table S3

Summary of binding affinities of different PHD fingers with methylated H3 tail peptides.

Protein	Peptide	K_D (μM)	Measuring method	Reference
PHF20	H3K4me0	73 ± 6	Tryptophan fluorescence	(1)
	H3K4me1	49 ± 2		
	H3K4me2	20 ± 6		
	H3K4me3	3.6 ± 0.6		
MLL5	H3K4me0	778 ± 183	NMR	(2)
	H3K4me1	319 ± 18		
	H3K4me2	104 ± 14	Tryptophan fluorescence	
	H3K4me3	21 ± 9		
SET3	H3K4me0	1400 ± 300	NMR	(3)
	H3K4me1	790 ± 15		
	H3K4me2	30 ± 5	Tryptophan fluorescence	
	H3K4me3	20 ± 7		
TAF3	H3K4me3	0.31 ± 0.09	Tryptophan fluorescence	(4)
ING2	H3K4me0	2240 ± 350	NMR	(5)
	H3K4me1	208 ± 80	Tryptophan fluorescence	
	H3K4me2	15 ± 4		
	H3K4me3	1.5 ± 1		
ING3	H3K4me0	180.62 ± 19.17	ITC	(6)
	H3K4me1	23.24 ± 0.80		
	H3K4me2	2.99 ± 0.33		
	H3K4me3	0.93 ± 0.04		

Reference

1. Klein BJ, Wang X, Cui G, Yuan C, Botuyan MV, Lin K, et al. PHF20 Readers Link Methylation of Histone H3K4 and p53 with H4K16 Acetylation. Cell reports. 2016;17(4):1158-70. doi: 10.1016/j.celrep.2016.09.056
2. Ali M, Rincón-Arano H, Zhao W, Rothbart SB, Tong Q, Parkhurst SM, et al. Molecular basis for chromatin binding and regulation of MLL5. Proceedings of the National Academy of Sciences of the United States of America. 2013;110(28):11296-301. doi: 10.1073/pnas.1310156110
3. Gatchalian J, Ali M, Andrews FH, Zhang Y, Barrett AS, Kutateladze TG. Structural Insight into Recognition of Methylated Histone H3K4 by Set3. Journal of molecular biology. 2017;429(13):2066-74. doi: 10.1016/j.jmb.2016.09.020
4. van Ingen H, van Schaik FM, Wienk H, Ballering J, Rehmann H, Dechesne AC, et al. Structural insight into the recognition of the H3K4me3 mark by the TFIID subunit TAF3. Structure (London, England : 1993). 2008;16(8):1245-56. doi: 10.1016/j.str.2008.04.015
5. Peña PV, Davrazou F, Shi X, Walter KL, Verkhusha VV, Gozani O, et al. Molecular mechanism of histone H3K4me3 recognition by plant homeodomain of ING2. Nature. 2006;442(7098):100-3. doi: 10.1038/nature04814
6. Kim S, Natesan S, Cornilescu G, Carlson S, Tonelli M, McClurg UL, et al. Mechanism of Histone H3K4me3 Recognition by the Plant Homeodomain of Inhibitor of Growth 3. The Journal of biological chemistry. 2016;291(35):18326-41. doi: 10.1074/jbc.M115.690651
